# Contagem Absoluta de Linfócitos: um Preditor de PCR Sérica Positiva para o *Trypanosoma cruzi* em Pacientes com Chagas Submetidos ao Transplante Cardíaco

**DOI:** 10.36660/abc.20230588

**Published:** 2024-06-26

**Authors:** Plínio José Whitaker Wolf, Marco Aurelio Finger, João Manoel Rossi, Carolina Casadei Santos, Victor Bemfica de Mello Mattos, Raphael Rossi, Lucas Petri Damiani

**Affiliations:** 1 Instituto Dante Pazzanese de Cardiologia São Paulo SP Brasil Instituto Dante Pazzanese de Cardiologia, São Paulo, SP – Brasil; 2 Hospital Israelita Albert Einstein São Paulo SP Brasil Hospital Israelita Albert Einstein – ARO, São Paulo, SP – Brasil

**Keywords:** Transplante de Coração, Doença de Chagas, Linfopenia, Terapia de Imunossupressão

## Abstract

**Fundamento:**

É desconhecido se a linfopenia é fator de risco para a reativação da doença de Chagas no transplante cardíaco (TxC), como recentemente descrito na reativação de citomegalovírus em pacientes transplantados.

**Objetivo:**

Avaliar se a linfopenia no perioperatório do TxC está relacionada à parasitemia precoce pelo *Trypanosoma cruzi*.

**Métodos:**

Amostra analisada (janeiro de 2014 a janeiro de 2023) em estudo observacional e retrospectivo. A parasitemia foi avaliada nos primeiros 3 meses após o TxC por meio da reação em cadeia da polimerase sérica (PCR) e comparada com a contagem total de linfócitos no perioperatório do TxC por curvas ROC. Comparadas características de base com a PCR Chagas por modelos de risco proporcionais de Cox independentes. Nível de significância adotado de 5%.

**Resultados:**

Amostra (n = 35) apresentou idade média de 52,5 ± 8,1 anos e PCR Chagas positiva em 22 pacientes (62,8%). As médias dos menores valores de linfócitos nos primeiros 14 dias do TxC foram 398 ± 189 e 755 ± 303 células/mm^3^ em pacientes com e sem parasitemia nos 3 meses após o TxC, respectivamente (área sob a curva = 0,857; intervalo de confiança de 95%: 0,996 a 0,718, sensibilidade e especificidade de 83,3% e 86,4%). Determinado valor de corte inferior a 550 linfócitos/mm^3^ como fator de risco para presença de parasitemia. Pacientes com linfócitos < 550 unidades/mm^3^ nos primeiros 14 dias do pós-TxC apresentaram PCR positiva em 80% dos casos. Para cada aumento de 100 linfócitos/mm^3^, o risco de positividade da PCR é reduzido em 26% (razão de riscos = 0,74; intervalo de confiança de 95%: 0,59 a 0,93, p = 0,009).

**Conclusão:**

Houve associação entre a linfopenia no perioperatório do TxC com a parasitemia precoce pelo *T. cruzi* detectada por PCR.

## Graphical abstract

**Figura Central:**
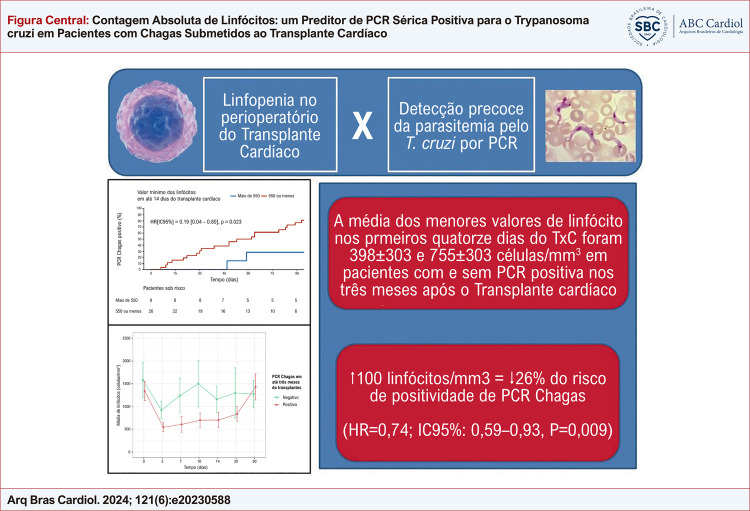
: Contagem Absoluta de Linfócitos: um Preditor de PCR Sérica Positiva para o Trypanosoma cruzi em Pacientes com Chagas Submetidos ao Transplante Cardíaco

HR: razão de riscos; IC: intervalo de confiança; PCR: reação em cadeia da polimerase; TxC: transplante cardíaco.

## Introdução

A doença de Chagas (DC) afeta, aproximadamente, 1,9 a 4,6 milhões de indivíduos no Brasil (1% a 2,4% da população),^1^ levando em consideração a elevada prevalência e incidência anual de insuficiência cardíaca,^2^ envolvendo, inclusive, países desenvolvidos, devido à atual globalização e consequente emigração de povos latino-americanos.^3,4^ A doença é causada pelo protozoário *Trypanosoma cruzi (T. cruzi)* e transmitida pelos insetos da família *Triatominae* (pela inoculação de suas fezes infectadas após sua picada, ou por via oral, através da ingestão acidental do inseto e/ou suas fezes), podendo ser também transmitida por vias vertical, transfusional, pelo transplante de órgãos contaminados ou acidentes com materiais biológicos. A DC, em sua forma crônica, pode acometer órgãos específicos em 30% a 40% dos infectados, expressando-se nas formas cardíacas, digestivas ou mistas,^5^ sendo a forma cardíaca a mais comum, manifestada por sintomas de insuficiência cardíaca, angina, tromboembolismo, arritmias ou mesmo morte súbita.^4^ Em pacientes de alto risco (segundo escore de RASSI), o prognóstico é desfavorável, podendo alcançar uma mortalidade de 84% em 10 anos.^6^

Em casos de miocardiopatia chagásica refratária, o transplante cardíaco (TxC) é uma importante opção terapêutica (terceira principal causa de TxC no Brasil), uma vez que o prognóstico desses pacientes, após o transplante, é favorável, por serem doentes jovens, com poucas comorbidades e baixas taxas de hipertensão pulmonar, doença vascular de enxerto e reoperação.^3,7^

A imunossupressão, por sua vez, apesar de ser imprescindível para redução da rejeição e, consequentemente, para o aumento da sobrevida após o TxC, pode levar a importantes complicações, como a reativação da doença de Chagas (RDC).^3,7^

Descrita inicialmente em pacientes com neoplasias hematológicas em 1980 e, posteriormente, em portadores do vírus da imunodeficiência humana (HIV) em 1990,^8,9^ a RDC ocorre em 26,4% a 40% dos transplantados por miocardiopatia chagásica,^9^ podendo chegar a 61%, conforme Gray et al. demonstraram.^10^ A condução adequada diante da RDC é essencial, devido à elevada morbimortalidade quando não tratada adequadamente. Contudo, quando é realizado o diagnóstico precoce e o tratamento correto é empregado (benzonidazol como primeira linha), a mortalidade encontrada é inferior a 1%.^3,7,9^

A RDC apresenta manifestações cardíacas e extracardíacas, sendo a miocardite, sintomática ou não, a mais comum.^3,4^ O diagnóstico se baseia na clínica associada à evidência do parasita nos tecidos, por métodos parasitológicos (pesquisa direta do *T. cruzi*), histológicos em biópsias endomiocárdicas (BEM) ou pelo teste de reação em cadeia de polimerase (PCR) no sangue (ou no material da biópsia),^3,7,9^ método este não invasivo, com alta precocidade diagnóstica e acurácia.^11,12^

Devido à alta prevalência e ao prognóstico desfavorável, a determinação de fatores de risco para a RDC é essencial. De forma semelhante ao Chagas, a reativação do citomegalovírus (CMV), infecção comum no transplante cardíaco relacionada à imunossupressão, tem a linfopenia como importante fator de risco no pós-transplante, conforme evidenciado em estudos recentes.^13-16^ Apesar de a resposta imunológica da DC, assim como no CMV, também ser intimamente dependente dos linfócitos,^7,17^ não há estudos que avaliaram a linfopenia como fator de risco para a RDC até a presente análise, cujo objetivo é avaliar a relação entre a contagem absoluta de linfócitos no perioperatório do TxC e a detecção sérica precoce do *T. cruzi* por PCR, auxiliando no diagnóstico, rastreio e tratamento precoces da RDC.

## Métodos

Trata-se de um estudo observacional, retrospectivo, unicêntrico, realizado pela coleta e análise de dados de prontuário físico/eletrônico dos pacientes com cardiopatia chagásica refratária e que foram submetidos ao TxC no Instituto Dante Pazzanese de Cardiologia (IDPC).

### Seleção da amostra

Os pacientes selecionados para a amostra foram aqueles com insuficiência cardíaca avançada refratária (confirmada durante a avaliação prévia do TxC), secundária à cardiopatia chagásica (diagnosticada por dois testes sorológicos com métodos diferentes), que tinham indicação e que foram submetidos ao TxC no IDPC, no período de janeiro de 2014 a janeiro de 2023.

Foram selecionados pacientes que apresentaram sobrevida de pelo menos 30 dias do pós-operatório de TxC, sendo excluídos os indivíduos que vieram a óbito antes desse período.

**Figura 1:**
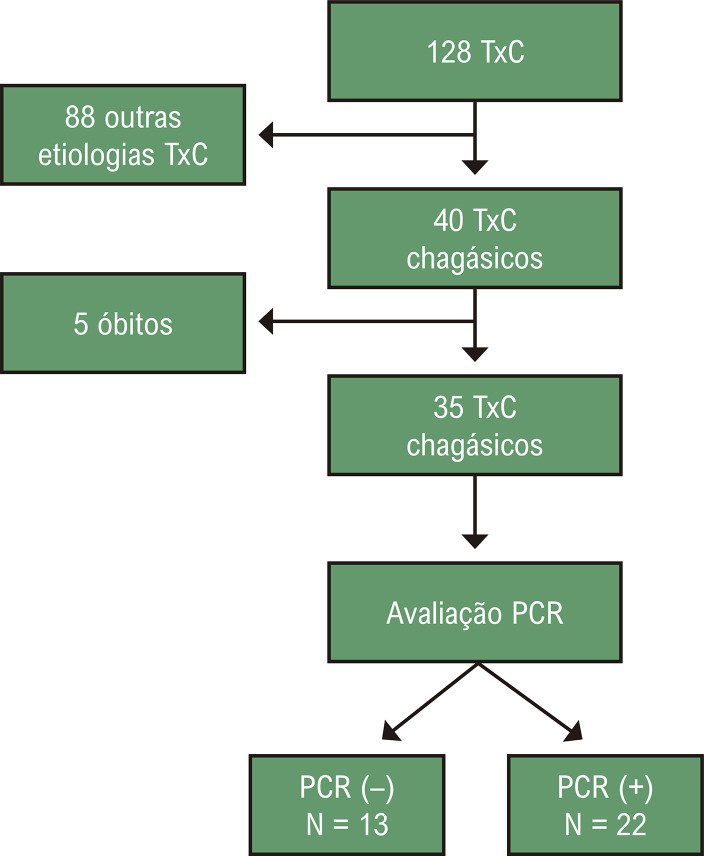
– Seleção da amostra do estudo. PCR: reação em cadeia da polimerase; TxC: transplante cardíaco.

De janeiro de 2014 a janeiro de 2023, foram realizados 128 TxC, dos quais 40 eram portadores da DC, confirmados por dois métodos diferentes. Dentre eles, 5 foram a óbito antes de 30 dias (nenhum óbito relacionado à RDC), resultando em uma amostra total de 35 pacientes, cuja PCR qualitativa para Chagas foi realizada, avaliando se houve detecção sérica do *T. cruzi* após o TxC (Figura 1).

### Definições e seleção de dados

A detecção sérica da parasitemia pelo *T. cruzi,* neste estudo, se deu por meio da PCR Chagas, analisada no Instituto Adolfo Lutz, a partir da amostra de sangue periférico, de forma qualitativa convencional, detectando DNA do *Trypanosoma cruzi* por meio da amplificação específica de um fragmento de 330 pb correspondente à região variável do minicírculo do kDNA. Foi avaliada, neste estudo, a positividade da PCR Chagas até o terceiro mês do TxC (período inicial do TxC, marcado por intensa imunossupressão e, portanto, maior incidência de RDC, conforme demonstraram Diez et al. com tempo médio de RDC avaliado em 71 dias).^12^ A frequência de solicitação da PCR Chagas seguiu o protocolo do serviço e, neste período estudado (primeiros 3 meses após TxC), foi solicitada pelo menos uma coleta até o primeiro mês e outra até o terceiro mês ou a qualquer momento na suspeita de RDC (alterações elétricas, ecocardiográficas ou clínicas sugestivas da reativação). Não existe um protocolo universal recomendado para solicitação de PCR e, por isso, os protocolos devem ser adequados às características e disponibilidades de cada serviço.^3^

A contagem absoluta de linfócitos foi avaliada pelo hemograma completo de sangue periférico (método citoquímico/isovolumétrico) do laboratório Associação Fundo de Incentivo à Pesquisa (AFIP), imediatamente antes do TxC (D0) e nos seguintes momentos do pós-operatório (PO): três dias, sete dias, dez dias, quatorze, trinta e noventa dias (D3, D7, D10, D14, D30 e D90, respectivamente). Os intervalos D0 e D7 foram baseados em estudos prévios que avaliaram a linfopenia na reativação do CMV,^14,16^ e os demais intervalos foram selecionados por opção dos autores. A disponibilidade desses dados foi possível devido à rotina do serviço, em que ocorre a solicitação de exames laboratoriais durante todo o perioperatório do TxC (e, após, nos retornos ambulatoriais), incluindo o hemograma completo, no pré-operatório e, diariamente, no PO de tal forma que todos os pacientes apresentaram adequadamente a dosagem de linfócitos nesses períodos. Diante de ampla disponibilidade de dados disponíveis em relação à contagem de linfócitos, o valor dos linfócitos coletado e utilizado no estudo foi exatamente o dos dias determinados: D0, D3, D7, D10, D14, D30 e D90.

Foi realizada a média dos menores valores de linfócitos totais encontrados nos primeiros quatorze dias do TxC e analisada de acordo com a reatividade da PCR Chagas. Utilizou-se o menor valor dos linfócitos para avaliar a hipótese de que a linfopenia possa estar associada à detecção sérica do *T. cruzi* e à RDC.

Evidente que, como sabidamente se espera na pluralidade dos TxC precoces, a maioria dos pacientes incluídos sofreu alguma mudança no esquema imunossupressor (dose e tipo) durante o seguimento, alterado em casos de rejeição, infecção ou efeitos adversos (lesão renal aguda, por exemplo), mas o estudo não avaliou a variação da imunossupressão e seu possível impacto na contagem de linfócitos. Assim, a terapia imunossupressora foi avaliada em torno do terceiro mês do pós-transplante, quando o tipo e as doses dos imunossupressores tendem a se estabilizar e as biópsias já foram realizadas para definição do grau de imunossupressão. Além disso, esse período é próximo ao tempo médio da RDC de 71 dias após TxC sugerido por Diez et al.^12^

A pesquisa de CMV também foi previamente realizada nos pacientes e analisada neste estudo, dentro do período de 3 meses após TxC, de forma preemptiva, conforme protocolo institucional (2, 6 e 10 semanas), por meio da PCR quantitativa, realizada pelo laboratório AFIP (detecção a partir de 50 cópias/mL).

Demais dados selecionados foram aqueles relacionados ao perfil clínico e laboratorial e anatomopatológico (pela BEM) do paciente, extraídos do prontuário.

Foram coletadas características basais dos pacientes, como idade, sexo, tipagem sanguínea, peso, altura, índice de massa corpórea (IMC), data da cirurgia, ocorrência de óbito (se presente), além das comorbidades que se apresentavam previamente ao TxC, como hipertensão arterial sistêmica, diabetes mellitus, dislipidemia e histórico de tabagismo.

Ainda, foram avaliados na amostra a presença e o grau de rejeição celular (classificação conforme proposto por Stewart et al., em 2005^18^) pela BEM realizada conforme protocolo institucional (segunda, quarta, oitava e décima-segunda semanas após o TxC).

### Análise estatística

As variáveis contínuas foram descritas por média ± desvio-padrão ou mediana e intervalo interquartil, conforme normalidade de dados, avaliadas através da inspeção por histogramas. As variáveis categóricas foram apresentadas por suas frequências absolutas e relativas.

As comparações das características de base em relação ao resultado de PCR Chagas, nos 3 meses do TxC, foram realizadas por modelos de riscos proporcionais de Cox independentes. Os resultados foram apresentados por razões de taxa de risco com intervalos de confiança de 95%.

Foi avaliada a associação entre a contagem absoluta de linfócitos no perioperatório (por meio da média do menor valor dos linfócitos em até 7 e 14 dias de PO) e a presença da PCR Chagas positiva em até três meses do TxC, a partir de curvas ROC. As áreas sob as curvas (AUC) foram descritas para cada caso.

Três modelos de tempo até evento, considerando modelos de riscos proporcionais de Cox, foram apresentados para mostrar a chance de positivar o resultado da PCR para Chagas de acordo com os valores de linfócitos de base (D0), e mínimo até 7 dias e até 14 dias do PO. Modelos de sensibilidade ajustados (por idade, dose de micofenolato e episódios de rejeição com indicação de pulsoterapia) também foram incluídos. Construiu-se um gráfico de Kaplan-Meier para a avaliação dos linfócitos de melhor acurácia.

As análises foram conduzidas com auxílio do software R, versão 4.2.1. Testes de hipótese foram avaliados ao nível de significância de 5%.

## Considerações éticas

O estudo atendeu a todos os princípios éticos requeridos, tendo sido encaminhado e aprovado, primeiramente, pelo Comitê de Ética em Pesquisa do Instituto Dante Pazzanese (número de protocolo do Comitê de Ética: 5331; número do parecer: 5918442; CAAE: 67067923.3.0000.5462), e esteve em conformidade com a lei geral de proteção de dados e resolução 466/2012.

## Resultados

O estudo apresentou amostra total de 35 pacientes, idade média de 52,5 ± 8,1 anos, com IMC médio de 22,6 ± 2,8 kg/m^2^. Durante o seguimento de 3 meses, houve 4 óbitos, 11% da amostra, que não apresentaram relação com RDC. A Tabela 1 sumariza as características de base, de acordo com a análise da PCR Chagas. Não houve associação das variáveis descritas com a positividade da PCR, incluindo tipo de imunossupressor, doses mais elevadas do micofenolato, episódios de rejeição (> 2R com indicação de pulsoterapia) ou reativação/carga viral de CMV.

**Tabela 1:** – Comparação das características de base de acordo com a positividade da PCR Chagas em até 3 meses do pós-operatório do transplante cardíaco (n = 35)

Variáveis	Total	Negativo, N = 13	Positivo, N = 22	Razão de taxa de riscos [IC95%]	p*
Idade, anos, média ± DP	52,5 ± 8,1	50,8 ± 8,8	53,5 ± 7,7	1.03 [0,97 –1,08]	0,358
Sexo masculino, n/N (%)	22/35 (62,9%)	6/13 (46,2%)	16/22 (72,7%)	1,51 [0,63 – 3,65]	0,358
**Tipagem sanguínea, n/N (%)**					
A	11/35 (31,4%)	5/13 (38,5%)	6/22 (27,3%)	referência	
B	5/35 (14,3%)	2/13 (15,4%)	3/22 (13,6%)	1,43 [0,4 – 5,09]	0,579
AB	6/35 (17,1%)	1/13 (7,7%)	5/22 (22,7%)	2,1 [0,62 – 7,07]	0,232
O	13/35 (37,1%)	5/13 (38,5%)	8/22 (36,4%)	1,11 [0,39 – 3,13]	0,841
HAS, n/N (%)	11/35 (31,4%)	4/13 (30,8%)	7/22 (31,8%)	0,74 [0,31 – 1,8]	0,509
DM, n/N (%)	5/35 (14,3%)	2/13 (15,4%)	3/22 (13,6%)	0,64 [0,19 – 2,17]	0,474
DLP, n/N (%)	13/35 (37,1%)	5/13 (38,5%)	8/22 (36,4%)	0,7 [0,31 – 1,62]	0,408
Ex-tabagista, n/N (%)	12/35 (34,3%)	3/13 (23,1%)	9/22 (40,9%)	1,52 [0,66 – 3,5]	0,324
**Número de imunossupressores no PO 3 meses, n/N (%)**					
2	1/35 (2,9%)	0/13 (0,0%)	1/22 (4,5%)	referência	
3	33/35 (94,3%)	13/13 (100,0%)	20/22 (90,9%)	0,31 [0,04 – 2,47]	0,270
4	1/35 (2,9%)	0/13 (0,0%)	1/22 (4,5%)	0,45 [0,03 – 7,61]	0,582
Micofenolato, n/N (%)	34/35 (97,1%)	13/13 (100,0%)	21/22 (95,5%)	0,32 [0,04 – 2,5]	0,276
Dose > 1000 mg/dia mofetil ou > 720 mg/dia sódico, n/N (%)	4/35 (11,4%)	0/13 (0,0%)	4/22 (18,2%)	1,75 [0,58 – 5,25]	0,321
Azatioprina, n/N (%)	0/35 (0,0%)	0/13 (0.0%)	0/22 (0.0%)	-	-
Tacrolimus, n/N (%)	15/35(42,9%)	3/13 (23,1%)	12/22 (54,5%)	1,51 [0,67 – 3,36]	0,318
Ciclosporina, n/N (%)	20/35 (57,1%)	10/13 (76,9%)	10/22 (45,5%)	0,66 [0,3 – 1,48]	0,318
Prednisona, n/N (%)	35/35 (100,0%)	13/13 (100,0%)	22/22 (100,0%)	-	-
Inibidor mTOR, n/N (%)	1/35 (2,9%)	0/13 (0,0%)	1/22 (4,5%)	1,4 [0,19 – 10,57]	0,743
Reativação de CMV, n/N (%)	25/35 (71,4%)	8/13 (61,5%)	17/22 (77,3%)	1,54 [0,57 – 4,13]	0,391
Logaritmo da carga viral máxima de CMV, log (cópia/ml), mediana [quartis]	7,3 [6,6; 9,0]	8,9 [7,3; 9,9]	8,6 [6,7; 9,9]	1,05 [0,89 – 1,24]	0,574
Rejeição > 2R/pulsoterapia, n/N (%)	19/35 (54,3%)	7/13 (53,8%)	12/22 (54,5%)	1,21 [0,53 – 2,73]	0,650

*Modelos de riscos proporcionais de Cox para PCR negativa em até 3 meses pós transplante. CMV: citomegalovírus; DLP: dislipidemia; DM: diabetes mellitus; DP: desvio-padrão; HAS: hipertensão arterial; IC: intervalo de confiança; mTOR: proteína alvo da rapamicina em mamíferos; PCR: reação em cadeia de polimerase qualitativa; PO: pós-operatório; TxC: transplante cardíaco.

No período de até 3 meses após o TxC, 22 pacientes apresentaram PCR Chagas positiva, ou seja, 62% da amostra, sendo que o tempo médio para a reatividade da PCR foi de 45 dias ± 26,2. Todos os pacientes que apresentaram PCR Chagas positiva receberam tratamento com benzonidazol por 60 dias, conforme protocolo do serviço, sendo que todos eles negativaram PCR após o tratamento e nenhum apresentou complicações ligadas à RDC após. Não houve outros pacientes que receberam benzonidazol, além daqueles que apresentaram PCR positiva.

**Figura 2:**
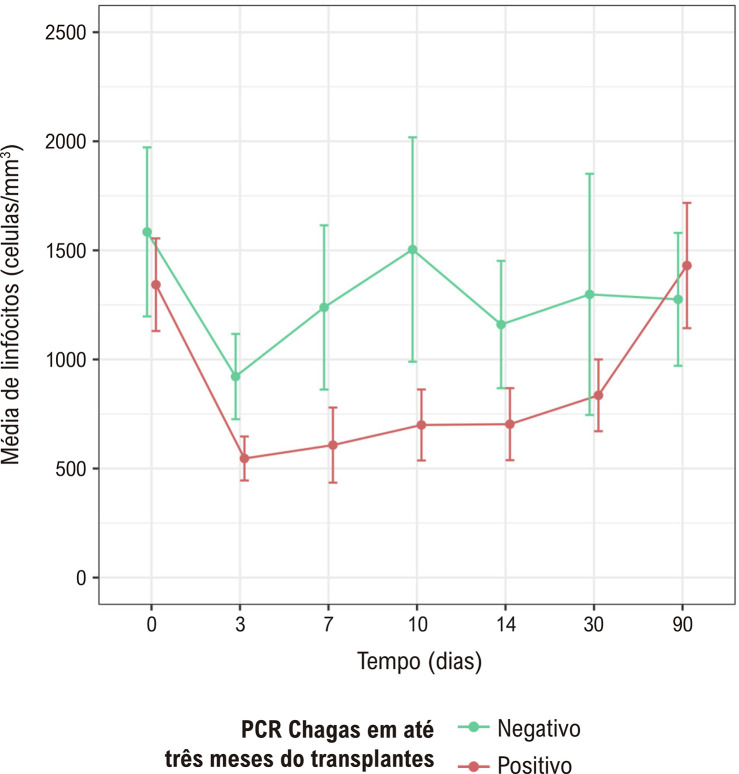
– Média e desvio padrão dos linfócitos por dia, segundo resultados da PCR Chagas (até 3 meses do transplante cardíaco). PCR: reação em cadeia da polimerase.

**Figura 3:**
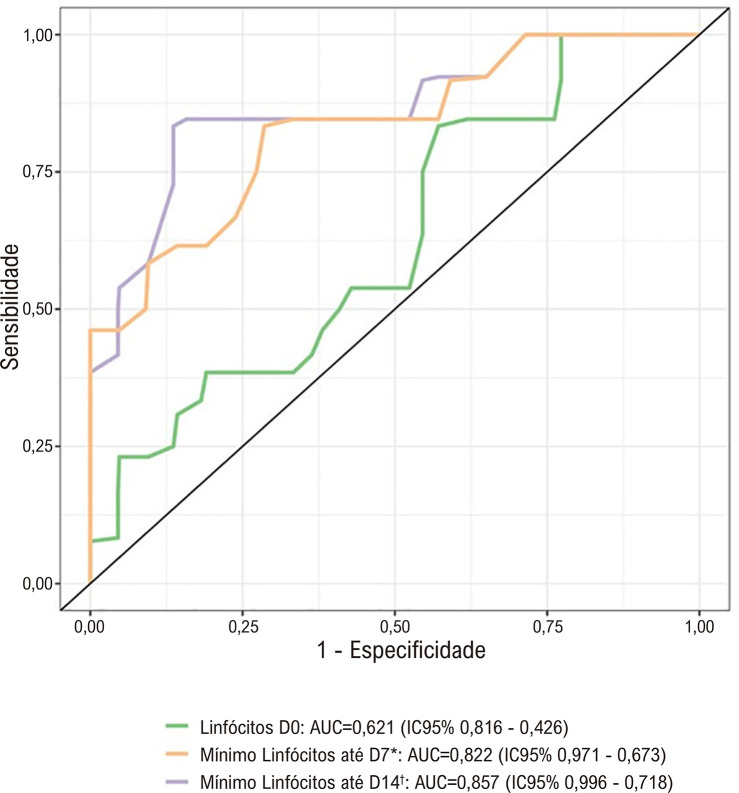
– Curvas ROC para a média dos linfócitos de base (pré-operatório imediato, D0) e a média dos menores valores de linfócitos em até 7 dias (D7) e até 14 dias (D14) após transplante como preditor de negatividade para PCR Chagas em até 3 meses do transplante cardíaco. *Considerando a média dos menores valores dos linfócitos nas avaliações até D7 (incluindo D0, D3 e D7). †Média dos menores valores de linfócitos nas avaliações até D14 (incluindo D0, D3, D7, D10 e D14). AUC: área abaixo da curva ROC; D: dia após o transplante cardíaco; IC: intervalo de confiança.

A Tabela 2 e a Figura 2 descrevem os valores de linfócitos de acordo com o resultado da PCR Chagas até o terceiro mês do TxC. A Tabela 2, ainda, apresenta a AUC dos linfócitos para a positividade da PCR para *T. cruzi* (representado graficamente pela Figura 3), além dos pontos de corte sugeridos como fator de risco (para reatividade da PCR) para os valores do linfócito de base (D0), do mínimo em até D7 e do mínimo em até D14.

**Tabela 2:** – Média e desvio padrão da contagem absoluta de linfócitos por dia, segundo resultados da PCR Chagas em até 3 meses do transplante cardíaco, com AUC e ponto de corte de linfócitos como fator de risco para reatividade de PCR

Dia (D)	PCR Chagas em até 3 meses do TxC			Ponto de corte ^**‡**^	Sensibilidade	Especificidade

	Negativo	Positivo	AUC			
D0	1585 ± 713	1343 ± 508	0,621	1120	83,3%	42,9%
D3	922 ± 360	546 ± 241	0,808			
D7	1238 ± 693	607 ± 412	0,795			
D10	1504 ± 946	700 ± 390	0,778			
D14	1160 ± 537	703 ± 395	0,771			
Mínimo* em 7 dias	810 ± 342	438 ± 224	0,822	550	83,3%	71,4%
Mínimo† em 14 dias	755 ± 303	398 ± 189	0,857	550	83,3%	86,4%

*Considerando a média dos menores valores dos linfócitos nas avaliações até D7 (incluindo D0, D3 e D7). †Média dos menores valores de linfócitos nas avaliações até D14 (incluindo D0, D3, D7, D10 e D14). ‡Linfócitos/mm^3^. AUC: área sob a curva ROC; PCR: reação em cadeia da polimerase; TxC: transplante cardíaco.

Nota-se que houve associação entre a média dos menores valores de linfócitos observados nos primeiros 7 e 14 dias do TxC e a reatividade da PCR Chagas ocorrida nos primeiros 3 meses, destacando-se a média dos menores valores de linfócitos encontrados nos primeiros 14 dias após o transplante (incluindo D3, D7, D10 e D14), que apresentou estatísticas preditoras (pela AUC) de maior relação com a positividade da PCR em até 3 meses do TxC. Neste caso, a média do valor mínimo de linfócitos foi de 755 ± 303 unidades/mm^3^ nos pacientes com PCR negativa, bastante superior ao valor médio de 398 ± 189 unidades/mm^3^ nos pacientes com PCR positiva nos primeiros 3 meses pós TxC, configurando AUC = 0,857. A partir desses dados, o ponto de corte como fator de risco para reatividade de PCR Chagas foi determinado em 550 linfócitos/mm^3^.

Destaca-se, na Figura 2, que a média de linfócitos nos pacientes com PCR positiva após o TxC é superior àqueles com PCR negativa, sobretudo nos períodos de 3 dias a 14 dias após o transplante, sendo os valores de linfócitos semelhantes após 90 dias.

Ainda, foi realizada a avaliação do tempo até positividade da PCR Chagas a partir de uma análise de sobrevivência, conforme demonstrado na Tabela 3. Considerando a média dos menores valores de linfócitos em até 7 e 14 dias, houve efeitos significativos para o aumento de cada 100 unidades de linfócitos. Para o valor mínimo até 14 dias, evidencia-se que, a cada 100 unidades a mais de linfócitos, o risco de reativação se reduz em 26% (razão de riscos = 0.74 [intervalo de confiança 95%: 0.59 a 0,93]). Ajustou-se o modelo a outras variáveis (idade, dose da imunossupressão com micofenolato e rejeição com indicação de pulsoterapia, esses dois últimos previamente descritos como relacionados à RDC^19^), mantendo significância estatística. Demais variáveis não foram utilizadas para ajustar o modelo devido à ausência de associação com a reatividade da PCR, conforme visto na Tabela 1.

**Tabela 3:** – Modelos de Cox para tempo até reativação segundo linfócitos contínuos

Modelo para tempo até reativação	Razão de taxa de riscos [IC95%]	Valor p	Razão de taxa de riscos ajustada‡ [IC95%]	Valor p
Linfócito pré-operatório imediato (D0)	0,79 [0,66 – 0,93]	0,006	0,75 [0,61 – 0,92]	0,007
Mínimo linfócitos até D7*	0,74 [0,59 – 0,93]	0,009	0,73 [0,58 – 0,93]	0,010
Mínimo linfócitos até D14†	0,74 [0,59 – 0,93]	0,009	0,73 [0,58 – 0,93]	0,010

*Considerando a média dos menores valores dos linfócitos nas avaliações até D7 (incluindo D0, D3 e D7). †Média dos menores valores de linfócitos nas avaliações até D14 (incluindo D0, D3, D7, D10 e D14). ‡ Modelos ajustados por idade, número de episódios de rejeição > 2R/pulsoterapia e doses mais elevadas de micofenolato (> 1000 mg/dia mofetil ou > 720 mg/dia sódico). D: dia após o transplante cardíaco; IC: intervalo de confiança.

**Figura 4:**
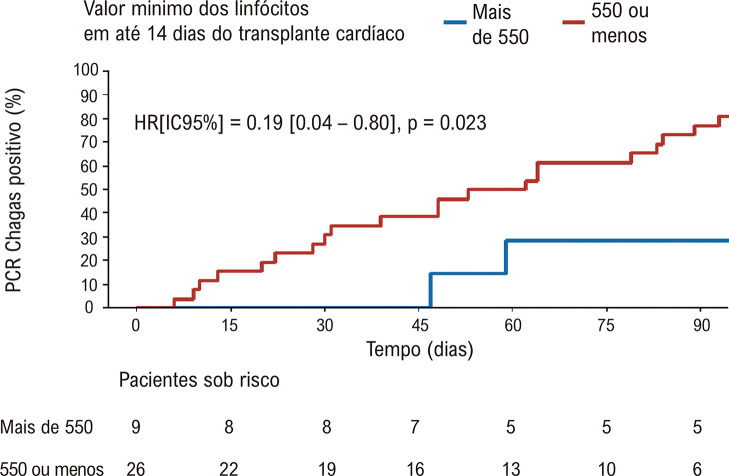
– Kaplan-Meier para positividade de PCR Chagas de acordo com o valor mínimo de linfócitos menor ou igual a 550 linfócitos/mm3, apresentado em até 14 dias do pós-operatório do transplante cardíaco.

Na Figura 4, percebe-se que os pacientes que alcançaram valores de linfócitos inferiores a 550 unidades/mm^3^, no pós-transplante, apresentaram PCR positivo em 80% dos casos precocemente, enquanto que portadores de linfócitos mínimos superiores a 550 unidades/mm^3^ positivaram PCR em apenas 30%, de forma mais tardia.

## Discussão

O TxC em pacientes com miocardiopatia chagásica refratária é frequente e apresenta prognóstico favorável, mas tem como complicação a RDC grave secundária à imunossupressão. Não se sabe se a linfopenia tem relação com a RDC, conforme recentemente descrito na reativação do CMV após o TxC.^13-16^

### PCR qualitativa para Chagas e a RDC

O estudo avaliou a reatividade da PCR Chagas e sua associação com a linfopenia no perioperatório do TxC. É sabido que a PCR qualitativa apresenta alta associação com o diagnóstico de RDC, conforme demonstraram Da Costa *et al*. em 2017, uma vez que esse exame apresenta sensibilidade acima de 80% para RDC, conferindo AUC = 0,702, o que é superior aos métodos diagnósticos convencionais para a RDC, além de conferir precocidade na identificação dessa complicação (em até 2 meses).^11^ Portanto, é razoável que a associação da linfopenia no perioperatório com a reatividade da PCR Chagas possa ter também relação com a RDC propriamente dita. A PCR sérica quantitativa, por sua vez, não foi utilizada neste estudo e já é conhecido que não existem valores de corte específicos para definição de RDC, pois, conforme propõem Benvenuti et al., o sangue nem sempre é capaz de refletir o grau de acometimento de outros órgãos pelo *T. cruzi*, como o miocárdio.^19,20^

A PCR Chagas pode ser positiva também em pacientes com miocardiopatia chagásica crônica avançada previamente ao TxC (imunocompetentes), conferindo a eles maior risco cardiovascular.^21^ Contudo, ainda não há consenso quanto ao tratamento etiológico desses pacientes,^5^ diferentemente dos pacientes submetidos ao TxC por DC com detecção do *T. cruzi* e RDC, em que o tratamento antiparasitário é fortemente recomendado.^3^ Ou seja, a monitorização com a PCR pode alterar a conduta nos transplantados portadores de DC, devido à possibilidade do diagnóstico e tratamento precoces.

### Linfopenia como fator de risco para parasitemia (detectada por PCR Chagas)

Inicialmente, a amostra apresentou, nos primeiros 3 meses após o TxC, PCR Chagas positiva em 62% dos pacientes, o que se assemelha à incidência de estudo norte-americano, em que a reativação alcançou a taxa de 61% dos transplantados,^10^ refletindo alta prevalência dessa complicação que, associada a sua gravidade, torna necessária a identificação dos seus fatores de risco para o manejo adequado da RDC.

Já são conhecidos alguns fatores de risco associados à RDC, como episódios de rejeição, presença de neoplasia e grau de imunossupressão com o micofenolato.^19^ Contudo, não se sabe se a linfopenia tem relação com a RDC.

Os resultados deste estudo, em que a média dos linfócitos nos pacientes que apresentaram PCR positiva se mostrou inferior àqueles cuja PCR foi negativa (Figura 2), sobretudo para a média dos menores valores dos linfócitos nos primeiros 14 dias após o transplante (Tabela 2, com médias de 398 ± 189 e 755 ± 303 células/mm^3^ nos pacientes com PCR positiva e negativa, respectivamente, com sensibilidade e especificidade superiores a 80%), evidencia que houve associação entre a linfopenia e a reatividade da PCR e, analogamente, com a RDC, de forma semelhante ao constatado nos estudos sobre CMV.^13-16^ Soma-se a este achado o fato de que, como demonstra a Tabela 3, a cada aumento de 100 linfócitos, há redução do risco de reatividade da PCR em 26%.

Ressalta-se, também, a semelhança entre o valor de corte como fator de risco para positividade de PCR Chagas identificado, neste estudo, de 550 unidades/mm^3^, como descrito em estudos prévios com reativação do CMV após TxC, entre 500 e 610 unidades/mm^3^,^14,15^ reforçando a dependência da resposta imunológica frente a essas infecções.

Foi observada precocidade na positividade da PCR, que ocorreu em média após 45 dias do TxC, período pouco inferior ao demonstrado por Diez *et al*., em torno de 71 dias.^12^ Quando o número de linfócitos foi superior a 550 unidades/mm^3^ nos primeiros 14 dias, houve, além de evidente menor prevalência da PCR Chagas positiva, um retardo no tempo para positividade desse exame.

### Imunossupressão e a detecção de T. cruzi precoce por PCR Chagas

Neste estudo, conforme demonstrou a Tabela 1, não houve associação estatisticamente significativa entre a reatividade da PCR com episódios de rejeição prévios, apesar dessa associação já ter sido relatada previamente,^19^ provavelmente devido às limitações deste estudo, sobretudo referentes ao tamanho amostral. Não houve relação também com o número, tipo e dose de imunossupressores. Ressalta-se o fato de que o serviço avaliado não faz uso de azatioprina como imunossupressor de escolha nos pacientes com DC (conforme sugerem alguns autores^3^), por isso, a ausência de pacientes com esse antiproliferativo na amostra, fazendo-se opção pelo micofenolato na menor dose possível, pela evidência de sua superioridade nos desfechos do TxC em geral.^22^

Em relação à resposta imunológica contra o protozoário, destaca-se, principalmente, a imunidade celular, por meio dos linfócitos T CD4, produtores de anticorpos líticos e citocinas (como intérferon gama), que auxiliam na destruição de formas intracelulares dos parasitas, além de algum papel dos linfócitos T CD8 neste processo.^17^ Com a imunossupressão no TxC, há redução função/número dessas células, criando um ambiente propício para a RDC.^7^

Como confirmação da importância da imunidade celular no controle da DC, sabe-se que, em pacientes com infecções pelo HIV e *T. cruzi* concomitantes, há elevada frequência (maior que 80%) de RDC quando linfócitos T CD4 são inferiores a 200 células/mm^3^

### Contribuições dos achados na prática médica

O conhecimento da linfopenia como fator de risco para reatividade da PCR Chagas pode propiciar um melhor manejo dos transplantados por miocardiopatia chagásica, visto que a dosagem de linfócitos é um exame laboratorial amplamente disponível, de baixo custo e de fácil interpretação que pode selecionar pacientes de alto risco para a RDC. Dessa maneira, esses indivíduos podem ter uma vigilância mais rigorosa quanto à RDC, propiciando diagnósticos e tratamentos precoces. Na verdade, o estudo confirmou a alta eficácia do tratamento da RDC com benzonidazol, haja vista que houve resolução completa da parasitemia (confirmada com nova PCR, negativa) após o tratamento adequado em todos os pacientes que apresentaram PCR Chagas positiva.

Por fim, esses pacientes, inclusive, podem se beneficiar de tratamentos profiláticos e/ou redução da intensidade de imunossupressão. Apesar de Rossi et al. demonstrarem em estudo retrospectivo que houve benefício na redução da incidência da RDC com o uso do benzonidazol profilático após TxC,^23^ atualmente, o tratamento profilático contra a RDC não é recomendado pelas atuais diretrizes, sobretudo devido aos efeitos adversos causados por esse tratamento.^3^ No entanto, a identificação de pacientes de alto risco com linfopenia pode direcionar a indicação mais precisa de uma possível profilaxia medicamentosa, mas, certamente, são necessários novos estudos para avaliar o possível benefício desse tipo de conduta.

### Limitações

Evidentemente, o estudo apresenta limitações inerentes ao fato de ser retrospectivo, unicêntrico, além do tamanho amostral reduzido e ausência de validações interna e externa.

Além disso, apresenta disponibilidade limitada da realização de exames da PCR qualitativa sérica. Por fim, não há disponibilidade, no serviço, de realização da PCR Chagas quantitativa sérica ou da PCR Chagas realizada em material obtido pela biópsia do miocárdio.

## Conclusão

O estudo demonstrou que houve relação entre a baixa contagem absoluta de linfócitos durante o perioperatório do TxC com a detecção sérica precoce do *T. cruzi* por PCR nos primeiros 3 meses após o TxC.

## References

[1] Brasil, Ministério da Saúde, Secretaria de Vigilância em Saúde (2022). Boletim Epidemiológico: Territorialização e Vulnerabilidade para Doença de Chagas Crônica.

[2] Rohde LEP, Montera MW, Bocchi EA, Clausell NO, Albuquerque DC, Rassi S (2018). Diretriz Brasileira de Insuficiência Cardíaca Crônica e Aguda. Arq Bras Cardiol.

[3] Bacal F, Marcondes-Braga FG, Rohde LEP, Xavier JL, Brito FS, Moura LAZ (2018). 3ª Diretriz Brasileira de Transplante Cardíaco. Arq Bras Cardiol.

[4] Andrade JP, Marin JÁ, Paola AA, Vilas-Boas F, Oliveira GM, Bacal F (2011). I Latin American Guidelines for the Diagnosis and Treatment of Chagas' Heart Disease: Executive Summary. Arq Bras Cardiol.

[5] Marin-Neto JA, Rassi A, Oliveira GMM, Correia LCL, Ramos AN, Luquetti AO (2023). SBC Guideline on the Diagnosis and Treatment of Patients with Cardiomyopathy of Chagas Disease - 2023. Arq Bras Cardiol.

[6] Rassi A, Rassi A, Little WC, Xavier SS, Rassi SG, Rassi AG (2006). Development and Validation of a Risk Score for Predicting Death in Chagas' Heart Disease. N Engl J Med.

[7] Moreira MDCV, Cunha-Melo JR (2020). Chagas Disease Infection Reactivation after Heart Transplant. Trop Med Infect Dis.

[8] (2006). Recommendations for Diagnosis, Treatment and Follow-up of the Trypanosoma Cruzi: Human Immunodeficiency Virus Co-infection. Rev Soc Bras Med Trop.

[9] Dias JC, Ramos AN, Gontijo ED, Luquetti A, Shikanai-Yasuda MA, Coura JR (2016). Brazilian Consensus on Chagas Disease, 2015. Epidemiol Serv Saude.

[10] Gray EB, La Hoz RM, Green JS, Vikram HR, Benedict T, Rivera H (2018). Reactivation of Chagas Disease Among Heart Transplant Recipients in the United States, 2012-2016. Transpl Infect Dis.

[11] Costa PA, Segatto M, Durso DF, Moreira WJC, Junqueira LL, Castilho FM (2017). Early Polymerase Chain Reaction Detection of Chagas Disease Reactivation in Heart Transplant Patients. J Heart Lung Transplant.

[12] Diez M, Favaloro L, Bertolotti A, Burgos JM, Vigliano C, Lastra MP (2007). Usefulness of PCR Strategies for Early Diagnosis of Chagas' Disease Reactivation and Treatment Follow-up in Heart Transplantation. Am J Transplant.

[13] Gardiner BJ, Nierenberg NE, Chow JK, Ruthazer R, Kent DM, Snydman DR (2018). Absolute Lymphocyte Count: A Predictor of Recurrent Cytomegalovirus Disease in Solid Organ Transplant Recipients. Clin Infect Dis.

[14] Yoon M, Oh J, Chun KH, Lee CJ, Kang SM (2021). Post-transplant Absolute Lymphocyte Count Predicts Early Cytomegalovirus Infection after Heart Transplantation. Sci Rep.

[15] Schoeberl AK, Zuckermann A, Kaider A, Aliabadi-Zuckermann A, Uyanik-Uenal K, Laufer G (2023). Absolute Lymphocyte Count as a Marker for Cytomegalovirus Infection after Heart Transplantation. Transplantation.

[16] Meesing A, Razonable RR (2018). Absolute Lymphocyte Count Thresholds: A Simple, Readily Available Tool to Predict the Risk of Cytomegalovirus Infection after Transplantation. Open Forum Infect Dis.

[17] Brodskyn CI, Barral M Netto, Brener Z, Andrade ZA, Barral M (2000). Trypanosoma Cruzi e Doença de Chagas.

[18] Stewart S, Winters GL, Fishbein MC, Tazelaar HD, Kobashigawa J, Abrams J (2005). Revision of the 1990 Working Formulation for the Standardization of Nomenclature in the Diagnosis of Heart Rejection. J Heart Lung Transplant.

[19] Campos SV, Strabelli TM, Amato V, Silva CP, Bacal F, Bocchi EA (2008). Risk Factors for Chagas' Disease Reactivation after Heart Transplantation. J Heart Lung Transplant.

[20] Benvenuti LA, Freitas VLT, Roggério A, Nishiya AS, Mangini S, Strabelli TMV (2021). Usefulness of PCR for Trypanosoma Cruzi DNA in Blood and Endomyocardial Biopsies for Detection of Chagas Disease Reactivation after Heart Transplantation: A Comparative Study. Transpl Infect Dis.

[21] Mendes VG, Rimolo L, Lima ACB, Ferreira RR, Oliveira LS, Nisimura LM (2023). Biomarkers and Echocardiographic Predictors of Cardiovascular Outcome in Patients with Chronic Chagas Disease. J Am Heart Assoc.

[22] Hosenpud JD, Bennett LE (2001). Mycophenolate Mofetil Versus Azathioprine in Patients Surviving the Initial Cardiac Transplant Hospitalization: An Analysis of the Joint UNOS/ISHLT Thoracic Registry. Transplantation.

[23] Rossi JM, Finger MA, Santos CC (2020). Benznidazole as Prophylaxis for Chagas Disease Infection Reactivation in Heart Transplant Patients: A Case Series in Brazil. Trop Med Infect Dis.

