# Supplementary material for: Contagem Absoluta de Linfócitos: um Preditor de PCR Sérica Positiva para o Trypanosoma cruzi em Pacientes com Chagas Submetidos ao Transplante Cardíaco
Source: Arq Bras Cardiol. 2024 Jun 26;121(6):e20230588. [Article in Portuguese] doi: 10.36660/abc.20230588 (PMC11216328; doi:10.36660/abc.20230588)
Supplement: Translation

# Translation: Absolute Lymphocyte Count: a Predictor of Positive Serum PCR for Trypanosoma cruzi in Patients with Chagas Disease Undergoing Heart Transplantation

**DOI:** 10.36660/abc.20230588i

**Authors:** Plínio José Whitaker Wolf, Marco Aurelio Finger, João Manoel Rossi, Carolina Casadei Santos, Victor Bemfica de Mello Mattos, Raphael Rossi, Lucas Petri Damiani

**Affiliations:** 1 Brazil Instituto Dante Pazzanese de Cardiologia, São Paulo, SP – Brazil; 2 Brazil Hospital Israelita Albert Einstein – ARO, São Paulo, SP – Brazil

**Keywords:** Heart Transplantation, Chagas Disease, Lymphopenia, Immunosuppression Therapy

## Abstract

**Background:**

It is unknown whether lymphopenia is a risk factor for the reactivation of Chagas disease in heart transplantation (HTx), as recently described in the reactivation of cytomegalovirus in transplant patients.

**Objective:**

To evaluate whether lymphopenia in the perioperative period of heart transplantation is related to early Trypanosoma cruzi parasitemia.

**Methods:**

This observational, retrospective study analyzed a sample from January 2014 to January 2023). Parasitemia was evaluated in the first 3 months after HTx using serum polymerase chain reaction (PCR) and compared with the total lymphocyte count in the perioperative period of HTx using receiver operating characteristic curves. Baseline characteristics were compared with PCR for Chagas using independent Cox proportional hazards models. A significance level of 5% was adopted.

**Results:**

The sample (n = 35) had a mean age of 52.5 ± 8.1 years, and 22 patients (62.8%) had positive PCR for Chagas. The mean lowest lymphocyte values in the first 14 days after HTx were 398 ± 189 and 755 ± 303 cells/mm^3^ in patients with and without parasitemia, respectively, within 3 months after HTx (area under the curve = 0.857; 95% confidence interval: 0.996 to 0.718, sensitivity and specificity of 83.3% and 86.4%). A cutoff value of less than 550 lymphocytes/mm^3^ was determined as a risk factor for the presence of parasitemia. Patients with lymphocytes < 550 units/mm^3^ in the first 14 days after HTx presented positive PCR in 80% of cases. For every increase of 100 lymphocytes/mm^3^, the risk of PCR positivity was reduced by 26% (hazard rate ratio = 0.74; 95% confidence interval: 0.59 to 0.93, p = 0.009).

**Conclusion:**

There was an association between lymphopenia in the perioperative period of HTx and early T. cruzi parasitemia detected by PCR.

## Introduction

Chagas disease affects approximately 1.9 to 4.6 million individuals in Brazil (1% to 2.4% of the population),^1^ considering the high prevalence and annual incidence of heart failure.^2^ It affects developed countries as well, due to current globalization and consequent emigration of people from Latin American.^3,4^ The disease is caused by the protozoan *Trypanosoma cruzi* (*T. cruzi*), and it is transmitted by insects of the *Triatominae* family (through inoculation of infected feces after a bite or orally through accidental ingestion of the insect and/or its feces). It can also be transmitted vertically, by transfusion, through transplantation of contaminated organs, or accidents with biological materials. The chronic form of Chagas disease can affect specific organs in 30% to 40% of infected patients, expressed in cardiac, digestive, or mixed forms.^5^ The cardiac form is the most common, manifesting with symptoms of heart failure, angina, thromboembolism, arrhythmias, or even sudden death.^4^ In patients with high risks according to the RASSI score, the prognosis is unfavorable, reaching a mortality rate of 84% in 10 years.^6^

In cases of refractory Chagas cardiomyopathy, heart transplantation (HTx) is an important therapeutic option (third leading cause of HTx in Brazil), since the prognosis of these patients, after transplantation, is favorable, as they are young patients, with few comorbidities and low rates of pulmonary hypertension, graft vascular disease, and reoperation.^3,7^

Immunosuppression, in turn, although it is essential to reduce rejection and consequently increase survival after HTx, can lead to important complications, such as reactivation of Chagas disease (RCD).^3,7^

Initially described in patients with hematological neoplasms in 1980 and, subsequently, in patients with HIV in 1990,^8,9^ RCD occurs in 26.4% to 40% of patients undergoing transplantation due to Chagas cardiomyopathy,^9^ reaching 61%, as demonstrated by Gray et al.^10^ Appropriate management of RCD is essential, due to the high morbidity and mortality when not treated adequately. However, when it is diagnosed early and the correct treatment is used (benznidazole as first line), the mortality rate is less than 1%.^3,7,9^

RCD presents cardiac and extracardiac manifestations, with myocarditis, whether symptomatic or not, being the most common.^3,4^ Diagnosis is based on clinical examination associated with evidence of the parasite in the tissues, by means of parasitological methods (direct search for *T. cruzi*), histological analysis of endomyocardial biopsies, or polymerase chain reaction (PCR) test on blood (or biopsy material);^3,7,9^ the latter is a non-invasive method, with high diagnostic precocity and accuracy.^11,12^

Due to the high prevalence and unfavorable prognosis, it is essential to determine risk factors for RCD. Similar to Chagas, in reactivation of cytomegalovirus (CMV), a common infection in HTx related to immunosuppression, lymphopenia is an important risk factor in the post-transplant period, as shown in recent studies.^13-16^ Even though the immunological response of Chagas disease and CMV is also closely dependent on lymphocytes,^7,17^ there are no studies that have evaluated lymphopenia as a risk factor for RCD until the present analysis, whose objective was to evaluate the relationship between absolute lymphocyte count in the perioperative period of HTx and early serum detection of *T. cruzi* by PCR, assisting in early diagnosis, screening, and treatment of RCD.

## Methods

This observational, retrospective, single-center study was conducted by collecting and analyzing data from physical/electronic medical records of patients with refractory Chagas heart disease who underwent HTx at the Instituto Dante Pazzanese de Cardiologia (IDPC).

### Sample selection

The patients selected for this sample were those with advanced refractory heart failure (confirmed during prior HTx assessment), secondary to Chagas heart disease (diagnosed by 2 serological tests using different methods), who were indicated and who underwent HTx at IDPC, during the period from January 2014 to January 2023.

Patients who survived at least 30 days after HTx were selected, and patients who died before this period were excluded.

From January 2014 to January 2023, 128 HTx were performed, 40 of which were in patients with Chagas disease, confirmed by 2 different methods. Of these patients, 5 died before 30 days (no deaths related to RCD), resulting in a total sample of 35 patients, whose qualitative PCR for Chagas was performed to evaluate serum detection of *T. cruzi* after HTx (Figure 1).

**Figure 1:** – Study sample selection. HTx: heart transplantation; PCR: polymerase chain reaction.

### Definitions and data selection

In this study, serum detection of *T. cruzi* parasitemia was carried out using PCR for Chagas, analyzed at the Instituto Adolfo Lutz, using peripheral blood samples, in a conventional qualitative manner, detecting *Trypanosoma cruzi* DNA through specific amplification of a 330 bp fragment corresponding to the minicircle variable region of kDNA. In this study, PCR positivity for Chagas was evaluated up to the third month after HTx (initial period, marked by intense immunosuppression and, therefore, a higher incidence of RCD, as demonstrated by Diez et al. who observed a mean time to RCD of 71 days).^12^ The frequency of requesting PCR for Chagas followed the service protocol. In the period studied (first 3 months after HTx), at least one collection was requested by the first month and another by the third month or at any time when RCD was suspected (electrical, echocardiographic, or clinical changes suggestive of reactivation). There is no recommended universal protocol for requesting PCR; therefore, protocols must be adapted according to the characteristics and availability of each service.^3^

Absolute lymphocyte count was assessed by complete blood count of peripheral blood (cytochemical/isovolumetric method) at the Associação Fundo de Incentivo à Pesquisa (AFIP) laboratory, immediately before HTx (D0) and at the following postoperative moments: 3, 7, 10, 14, 30, and 90 days (D3, D7, D10, D14, D30, and D90, respectively). The D0 and D7 intervals were based on prior studies that evaluated lymphopenia in CMV reactivation,^14,16^ and the other intervals were selected at the authors’ discretion. These data were available due to the service’s routine, in which laboratory tests are requested throughout the perioperative period of HTx (and during outpatient follow-up), including complete blood count, in the preoperative period and daily during the postoperative period, so that all patients had adequate lymphocyte dosage during these periods. Given the wide availability of data available in relation to lymphocyte counts, the value of lymphocytes collected and used in the study was exactly that of the determined days: D0, D3, D7, D10, D14, D30, and D90.

The mean lowest values of total lymphocytes found in the first 14 days after HTx was obtained and analyzed according to PCR reactivity for Chagas. The lowest lymphocyte value was used to evaluate the hypothesis that lymphopenia may be associated with serum detection of *T. cruzi* and RCD.

It is evident that, as expected in numerous cases of early HTx, the majority of patients included underwent some change in the immunosuppressive regimen (dose and type) during follow-up, altered in cases of rejection, infection, or adverse effects (acute kidney injury, for example), but the study did not evaluate the variation in immunosuppression and its possible impact on lymphocyte counts. Thus, immunosuppressive therapy was evaluated around the third month after transplantation, when the type and doses of immunosuppressants tend to stabilize and biopsies have already been performed to define the degree of immunosuppression. Furthermore, this period is close to the mean time to RCD of 71 days after HTx suggested by Diez et al.^12^

A CMV test was also previously conducted for the patients and analyzed in this study, within a period of 3 months after HTx, preemptively, according to the institutional protocol (2, 6, and 10 weeks), by means of quantitative PCR, carried out at the AFIP laboratory (detection defined as 50 copies/mL).

Other selected data were related to patients’ clinical, laboratory, and anatomopathological profile (on endomyocardial biopsy), which were extracted from medical records.

Patients’ baseline characteristics were collected, including age, sex, blood type, weight, height, body mass index (BMI), date of surgery, occurrence of death (if present), in addition to comorbidities that were present prior to HTx, such as systemic arterial hypertension, diabetes mellitus, dyslipidemia, and history of smoking.

Moreover, the presence and degree of cellular rejection were assessed in the sample (using the classification proposed by Stewart et al., in 2005^18^) using endomyocardial biopsy performed according to the institutional protocol (second, fourth, eighth, and twelfth weeks after HTx).

### Statistical analysis

Continuous variables were described as mean ± standard deviation or median and interquartile range, according to data normality, evaluated through inspection of histograms. Categorical variables were shown as absolute and relative frequencies.

Comparisons of baseline characteristics in relation to the PCR results for Chagas, in the 3 months after HTx, were carried out using independent Cox proportional hazards models. Results were presented as hazard rate ratios with 95% confidence intervals.

The association between the absolute lymphocyte count in the perioperative period (by the mean lowest lymphocyte value up to 7 and 14 postoperative days) and the presence of positive PCR for Chagas within 3 months after HTx was evaluated using ROC curves. The areas under the curves (AUC) were described for each case.

Three time-to-event models, considering Cox proportional hazards models, were presented to show the chance of positive PCR results for Chagas according to the baseline lymphocyte values (D0) and minimum up to 7 days and up to 14 postoperative days. Adjusted sensitivity models (by age, mycophenolate dose, and rejection episodes with indication for pulse therapy) were also included. A Kaplan-Meier graph was constructed to evaluate the lymphocytes with better accuracy.

The analyses were conducted using R software, version 4.2.1. Hypothesis tests were performed at a 5% significance level.

### Ethical aspects

This study followed all required ethical principles. It was forwarded and approved, firstly, by the Research Ethics Committee of the Instituto Dante Pazzanese (ethics committee protocol number: 5331; opinion number: 5918442; CAAE: 67067923.3.0000.5462), and it was in compliance with the Brazilian General Data Protection Law and resolution 466/2012.

## Results

The study had a total sample of 35 patients, mean age of 52.5 ± 8.1 years, with mean BMI of 22.6 ± 2.8 kg/m^2^. During the 3-month follow-up, there were 4 deaths, 11% of the sample, which were not related to RCD. Table 1 summarizes the basic characteristics, according to the PCR analysis for Chagas. There was no association between the variables described and PCR positivity, including type of immunosuppressant, higher doses of mycophenolate, rejection episodes (> 2R with indication for pulse therapy), or CMV reactivation/viral load.

**Table 1:** – Comparison of baseline characteristics according to PCR positivity for Chagas within 3 postoperative months after heart transplantation surgery (n = 35)

Variables	Total	Negative, N = 13	Positive, N = 22	Hazard risk ratio [95% CI]	p*
Age, years, mean ± SD	52.5 ± 8.1	50.8 ± 8.8	53.5 ± 7.7	1.03 [0.97 –1.08]	0.358
Male sex, n/N (%)	22/35 (62.9%)	6/13 (46.2%)	16/22 (72.7%)	1.51 [0.63 – 3.65]	0.358
Blood type, n/N (%)					
A	11/35 (31.4%)	5/13 (38.5%)	6/22 (27.3%)	Reference	
B	5/35 (14.3%)	2/13 (15.4%)	3/22 (13.6%)	1.43 [0.4 – 5.09]	0.579
AB	6/35 (17.1%)	1/13 (7.7%)	5/22 (22.7%)	2.1 [0.62 – 7.07]	0.232
O	13/35 (37.1%)	5/13 (38.5%)	8/22 (36.4%)	1.11 [0.39 – 3.13]	0.841
SAH, n/N (%)	11/35 (31.4%)	4/13 (30.8%)	7/22 (31.8%)	0.74 [0.31 – 1.8]	0.509
DM, n/N (%)	5/35 (14.3%)	2/13 (15.4%)	3/22 (13.6%)	0.64 [0.19 – 2.17]	0.474
DLP, n/N (%)	13/35 (37.1%)	5/13 (38.5%)	8/22 (36.4%)	0.7 [0.31 – 1.62]	0.408
Ex-smoker, n/N (%)	12/35 (34.3%)	3/13 (23.1%)	9/22 (40.9%)	1.52 [0.66 – 3.5]	0.324
**Number of immunosuppressants in 3 PO months, n/N (%)**					
2	1/35 (2.9%)	0/13 (0.0%)	1/22 (4.5%)	Reference	
3	33/35 (94.3%)	13/13 (100.0%)	20/22 (90.9%)	0.31 [0.04 – 2.47]	0.270
4	1/35 (2.9%)	0/13 (0.0%)	1/22 (4.5%)	0.45 [0.03 – 7.61]	0.582
Mycophenolate, n/N (%)	34/35 (97.1%)	13/13 (100.0%)	21/22 (95.5%)	0.32 [0.04 – 2.5]	0.276
Dose > 1000 mg/day mycophenolate mofetil or > 720 mg/day mycophenolate sodium, n/N (%)	4/35 (11.4%)	0/13 (0.0%)	4/22 (18.2%)	1.75 [0.58 – 5.25]	0.321
Azathioprine, n/N (%)	0/35 (0.0%)	0/13 (0.0%)	0/22 (0.0%)	-	-
Tacrolimus, n/N (%)	15/35(42.9%)	3/13 (23.1%)	12/22 (54.5%)	1.51 [0.67 – 3.36]	0.318
Cyclosporine, n/N (%)	20/35 (57.1%)	10/13 (76.9%)	10/22 (45.5%)	0.66 [0.3 – 1.48]	0.318
Prednisone, n/N (%)	35/35 (100.0%)	13/13 (100.0%)	22/22 (100.0%)	-	-
mTOR inhibitor, n/N (%)	1/35 (2.9%)	0/13 (0.0%)	1/22 (4.5%)	1.4 [0.19 – 10.57]	0.743
Reactivation of CMV, n/N (%)	25/35 (71.4%)	8/13 (61.5%)	17/22 (77.3%)	1.54 [0.57 – 4.13]	0.391
Logarithm of maximum CMV viral load, log (copy/ml), median [quartiles]	7.3 [6.6; 9.0]	8.9 [7.3; 9.9]	8.6 [6.7; 9.9]	1.05 [0.89 – 1.24]	0.574
Rejection > 2R/pulse therapy, n/N (%)	19/35 (54.3%)	7/13 (53.8%)	12/22 (54.5%)	1.21 [0.53 – 2.73]	0.650

*Cox proportional hazards models for negative PCR within 3 months after transplantation. CI: confidence interval; CMV: cytomegalovirus; DLP: dyslipidemia; DM: diabetes mellitus; HTx: heart transplantation; mTOR: mammalian target of rapamycin protein; PCR: qualitative polymerase chain reaction; PO: postoperative; SAH: arterial hypertension; SD: standard deviation.

Within the period of 3 months after HTx, 22 patients had positive PCR for Chagas, that is, 62% of the sample, and the mean time to PCR reactivity was 45 ± 26.2 days. All patients who had positive PCR for Chagas received treatment with benznidazole for 60 days, according to the service protocol. They all had negative PCR results after treatment, and none of them presented complications linked to RCD afterwards. No other patients received benznidazole, besides those with positive PCR.

Table 2 and Figure 2 describe the lymphocyte values according to the PCR result for Chagas up to the third month after HTx. Table 2 also displays the AUC of lymphocytes for PCR positivity for *T. cruzi* (represented as a graph in Figure 3), in addition to the cutoff points suggested as a risk factor (for PCR reactivity) for baseline lymphocyte values (D0), the minimum by D7, and the minimum by D14.

**Table 2:** – Mean and standard deviation of absolute lymphocyte count per day, according to PCR results for Chagas within 3 months after heart transplantation, with AUC and cutoff point for lymphocytes as a risk factor for PCR reactivity

Day (D)	PCR for Chagas within 3 months after HTx			Cutoff point ‡	Sensitivity	Specificity

	Negative	Positive	AUC			
D0	1585 ± 713	1343 ± 508	0.621	1120	83.3%	42.9%
D3	922 ± 360	546 ± 241	0.808			
D7	1238 ± 693	607 ± 412	0.795			
D10	1504 ± 946	700 ± 390	0.778			
D14	1160 ± 537	703 ± 395	0.771			
Minimum* within 7 days	810 ± 342	438 ± 224	0.822	550	83.3%	71.4%
Minimum† within 14 days	755 ± 303	398 ± 189	0.857	550	83.3%	86.4%

* Considering the mean lowest lymphocyte values in assessments up to D7 (including D0, D3, and D7). † Mean lowest lymphocyte values in assessments up to D14 (including D0, D3, D7, D10, and D14). ‡ Lymphocytes/mm^3^. AUC: area under the receiver operating characteristic curve; HTx: heart transplantation; PCR: polymerase chain reaction.

**Figura 2:** – Mean and standard deviation of lymphocytes per day, according to PCR results for Chagas (up to 3 months after heart transplantation). PCR: polymerase chain reaction.

**Figure 3:** – ROC curves for mean baseline lymphocyte values (immediate preoperative period, D0) and mean lowest lymphocyte values within 7 days (D7) and 14 days (D14) after transplantation as a predictor of PCR negativity for Chagas within 3 months after heart transplantation. * Considering the mean lowest lymphocyte values in assessments up to D7 (including D0, D3, and D7). † Mean lowest lymphocyte values in assessments up to D14 (including D0, D3, D7, D10, and D14). AUC: area under the receiver operating characteristic curve; CI: confidence interval; D: day after heart transplantation.

There was an association between the mean lowest lymphocyte values observed in the first 7 and 14 days after HTx and PCR reactivity for Chagas that occurred in the first 3 months, highlighting the mean lowest lymphocyte values found in the first 14 days after transplantation (including D3, D7, D10, and D14), which presented predictive statistics (by AUC) of a greater relationship with PCR positivity within 3 months after HTx. In this case, the mean minimum value for lymphocytes was 755 ± 303 units/mm^3^ in patients with negative PCR, much higher than the mean value of 398 ± 189 units/mm^3^ in patients with positive PCR in the first 3 months after HTx, with AUC = 0.857. Based on these data, the cutoff point as a risk factor for PCR reactivity for Chagas was determined at 550 lymphocytes/mm^3^.

It is worth highlighting, in Figure 2, that the mean lymphocytes in patients with positive PCR after HTx was higher than in those with negative PCR, especially in periods from 3 to 14 days after transplantation, with lymphocyte values being similar after 90 days.

Furthermore, the time to PCR positivity for Chagas was evaluated based on survival analysis, as shown in Table 3. Considering the mean lowest lymphocyte values within 7 and 14 days, there were significant effects for each increase of 100 lymphocytes. For the minimum value up to 14 days, it is evident that, for each additional 100 units of lymphocytes, the risk of reactivation was reduced by 26% (hazard rate ratio = 0.74 [95% confidence interval: 0.59 to 0.93]). The model was adjusted for other variables (age, dose of immunosuppression with mycophenolate, and rejection with indication for pulse therapy, the latter two previously described as related to the RCD^19^), maintaining statistical significance. Other variables were not used to adjust the model due to the lack of association with PCR reactivity, as shown in Table 1.

**Table 3:** – Cox models for time to reactivation according to continuous lymphocytes

Model for time to reactivation	Hazard rate ratio [95%CI]	p value	Hazard rate ratio‡ [95%CI]	p value
Immediate preoperative lymphocyte (D0)	0.79 [0.66 – 0.93]	0.006	0.75 [0.61 – 0.92]	0.007
Minimum lymphocytes up to D7*	0.74 [0.59 – 0.93]	0.009	0.73 [0.58 – 0.93]	0.010
Minimum lymphocytes up to D14†	0.74 [0.59 – 0.93]	0.009	0.73 [0.58 – 0.93]	0.010

* Considering the mean lowest lymphocyte values in assessments up to D7 (including D0, D3, and D7). † Mean lowest lymphocyte values in assessments up to D14 (including D0, D3, D7, D10, and D14). ‡ Models adjusted for age, number of rejection episodes > 2R/pulse therapy and higher doses of mycophenolate (> 1000 mg/day mycophenolate mofetil or > 720 mg/day mycophenolate sodium). CI: confidence interval; D: day after heart transplantation.

Figure 4 shows that patients who had lymphocyte values below 550 units/mm^3^ after transplantation had positive PCR in 80% of cases early, whereas, in patients with minimum lymphocytes above 550 units/mm^3^, only 30% tested positive for PCR later.

**Figure 4:** – Kaplan-Meier for positive PCR for Chagas according to minimum lymphocyte value less than or equal to 550 lymphocytes/mm3, presenting within 14 postoperative days after heart transplantation. CI: confidence interval; HR: hazard rate ratio; PCR: polymerase chain reaction.

## Discussion

HTx in patients with refractory Chagas cardiomyopathy is common and has a favorable prognosis, but RCD secondary to immunosuppression is a severe complication. It is not known whether lymphopenia is related to RCD, as recently described in the reactivation of CMV after HTx.^13-16^

### Qualitative PCR for Chagas and reactivation of Chagas disease

The study evaluated PCR reactivity for Chagas and its association with lymphopenia in the perioperative period of HTx. It is known that qualitative PCR has a high association with diagnosis of RCD, as demonstrated by Da Costa et al. in 2017, since this test presents sensitivity greater than 80% for RCD, resulting in AUC = 0.702, which is superior to conventional diagnostic methods for RCD, in addition to providing early identification of this complication (within 2 months).^11^ Therefore, it is reasonable that the association of perioperative lymphopenia with PCR reactivity for Chagas may also be related to RCD itself. Quantitative serum PCR, in turn, was not used in this study, and it is already known that no specific cutoff values exist for defining RCD, since, as proposed by Benvenuti et al., blood is not always capable of reflecting the degree to which other organs, such as the myocardium, are affected by *T. cruzi*.^19,20^

PCR for Chagas may also be positive in patients with advanced chronic Chagas cardiomyopathy prior to HTx (immunocompetent patients), conferring a greater cardiovascular risk.^21^ However, there is still no consensus regarding the etiological treatment of these patients,^5^ unlike patients undergoing HTx for Chagas disease with detection of *T. cruzi* and RCD, for whom antiparasitic treatment is strongly recommended.^3^ In other words, monitoring with PCR can alter the management of transplant recipients with Chagas disease due to the possibility of early diagnosis and treatment.

### Lymphopenia as a risk factor for parasitemia (detected by PCR for Chagas)

Initially, the sample presented, within the first 3 months after HTx, positive PCR for Chagas in 62% of patients, which is similar to the incidence of a study from the United States, in which reactivation reached a rate of 61% in transplant recipients,^10^ reflecting the high prevalence of this complication, which, associated with its severity, makes it necessary to identify risk factors for the appropriate management of RCD.

Some risk factors associated with RCD are already known, such as episodes of rejection, presence of neoplasia, and degree of immunosuppression with mycophenolate.^19^ However, it is not known whether lymphopenia is related to RCD.

The results of this study, in which the mean number of lymphocytes in patients who had positive PCR was lower than those whose PCR was negative (Figure 2), especially for the mean lowest lymphocyte values in the first 14 days after transplantation (Table 2, with means of 398 ± 189 and 755 ± 303 cells/mm^3^ in patients with positive and negative PCR, respectively, with sensitivity and specificity greater than 80%), shows that there was an association between lymphopenia and PCR reactivity and, similarly, with RCD, similarly to the findings of studies on CMV.^13-16^ In addition to this finding, as shown in Table 3, for each increase of 100 lymphocytes, there was a 26% reduction in the risk of PCR reactivity.

It is also worth highlighting the similarity between the cutoff value identified in this study as a risk factor for PCR positivity for Chagas, namely, 550 units/mm^3^, and the one described in previous studies for CMV reactivation after HTx, between 500 and 610 units/mm^3^,^14,15^ reinforcing the dependence on the immune response when facing these infections.

Early PCR positivity was observed, which occurred a mean of 45 days after HTx, a period slightly shorter than that demonstrated by Diez et al., approximately 71 days.^12^ When the number of lymphocytes was greater than 550 units/mm^3^ in the first 14 days, in addition to an evidently lower prevalence of positive PCR for Chagas, there was a delay in the time to test positivity.

### Immunosuppression and early detection of T. cruzi by PCR

In this study, as shown in Table 1, there was no statistically significant association between PCR reactivity and previous episodes of rejection, although this association has previously been reported,^19^ probably due to the limitations of this study, especially regarding the sample size. There was also no relationship with the number, type, and dose of immunosuppressants. It is worth highlighting the fact that the service where the study was conducted does not use azathioprine as the immunosuppressant of choice in patients with Chagas disease (as suggested by some authors^3^); therefore, there were no patients using this antiproliferative drug in the sample, opting for the lowest possible dose of mycophenolate, due to the evidence of its superiority in HTx outcomes in general.^22^

In relation to the immunological response against the protozoan, cellular immunity stands out, mainly through CD4 T lymphocytes, producers of lytic antibodies and cytokines (such as gamma interferon), which assist in the destruction of intracellular forms of the parasites, in addition to some role of CD8 T lymphocytes in this process.^17^ With immunosuppression in HTx, there is a reduction in the function/number of these cells, creating an environment conducive to RCD.^7^

Regarding confirmation of the importance of cellular immunity in controlling Chagas disease, it is known that, in patients with concomitant HIV and *T. cruzi* infections, there is an elevated frequency (greater than 80%) of RCD when CD4 T lymphocytes are less than 200 cells/mm^3^

### Contributions of the findings to medical practice

Knowing that lymphopenia is a risk factor for PCR reactivity for Chagas can promote better management of patients undergoing transplantation due to Chagas cardiomyopathy, since lymphocyte measurement is a widely available, low-cost, and easy-to-interpret laboratory test that can select patients with high risk for RCD. Accordingly, these individuals can have more rigorous surveillance regarding RCD, providing early diagnoses and treatments. This study confirmed the high efficacy of treating RCD with benznidazole, given that there was complete resolution of the parasitemia (confirmed by a new negative PCR) after appropriate treatment in all patients who had positive PCR for Chagas.

Finally, these patients may even benefit from prophylactic treatments and/or reduction in the intensity of immunosuppression. Although Rossi et al., demonstrated in a retrospective study, that there was benefit in reducing the incidence of RCD with the use of prophylactic benznidazole after HTx,^23^ currently, prophylactic treatment against RCD is not recommended by current guidelines, especially due to the adverse effects caused by this treatment.^3^ Nonetheless, the identification of high-risk patients with lymphopenia can guide more precise indication of possible drug prophylaxis, but further studies are certainly needed to evaluate the possible benefit of this type of conduct.

### Limitations

Evidently, the study has limitations inherent to the fact that it is retrospective and single-center, in addition to the reduced sample size and lack of internal and external validation.

Furthermore, it presents limited availability to perform qualitative serum PCR tests. Finally, there was no availability, at the service, to perform quantitative serum PCR for Chagas or PCR for Chagas on material obtained from myocardial biopsy.

## Conclusion

This study demonstrated that there was a relationship between low absolute lymphocyte count during the perioperative period of HTx and early serum detection of *T. cruzi* by PCR in the first 3 months after HTx.
